# Supplementary material for: Community based hematological reference intervals among apparently healthy adolescents aged 12-17 years in Mekelle city, Tigrai, northern Ethiopia: A cross sectional study
Source: PLoS One. 2020 Sep 14;15(9):e0234106. doi: 10.1371/journal.pone.0234106 (PMC7489559; doi:10.1371/journal.pone.0234106)
Supplement: S1 Annexes — (DOCX) [file pone.0234106.s001.docx]

**Annexes**

**Aannex I: Informed assent / English version**

(The questionnaire is translated into the study participant's local language)

This informed assent form is for children apparently healthy individuals, who are invited to participate in this study on the reference interval of hematological parameters.

**Information Sheet**

**Introduction:** I am Hagos Haileslasie W/haweriat (BSc) studying medical hematology and Immunohematology at Addis Ababa University College of health science department of medical laboratory sciences. I am researching the reference interval. I am going to give you the information and invite you to be part of this research. There may be words that you do not understand. Please ask me to stop as we go through the information and I will take time to explain.

**Title of the project:** Community based hematological reference intervals among the apparently healthy adolescent’s aged 12-17 years in Mekelle city, Tigrai, North Ethiopia; A cross sectional study design from December 2018- May 2019

**Introduction:** You are kindly invited to participate on the establishment of hematological reference intervals among the apparently healthy individuals in Mekelle City, Tigrai, and Northern Ethiopia.

**Purpose of the study:** The purpose of the study is to establish community based hematological reference intervals which is important to the local population to screen for physiological or pathological conditions in routine health assessment

**Duration**: the duration of this study depend on the availability of study subjects and it can take about 3-5 months. However, specimen from you is collected only once.

**Procedures to be carried on**: you are invited to participate in the study after giving your consent by giving blood samples to assess your hematological profiles.

**Risks and Discomfort**: There will be minor discomfort or feel pain during collection of samples. During collection of samples from your hand appropriate precaution will be taken and all samples will be collected by trained health professionals. Appropriate medical care will be provided to you if needed.

**Expected Benefit**: the result of the study will be have direct benefit to you since you will be communicate with your results but there is no any financial benefit to you.

**Confidentiality:** Your name will not be written in the questioner and I assure that all the information you give and the laboratory results will be kept strictly confidential and could only be accessed by the researcher.

**Termination of the study:** The participation is based on your voluntary. You can resign participating in the study at any time. This decision will not affect in any way your current or future medical care in any health facility.

**Agreement**

After communicated in detail with guardians/parents about the study procedures and other related issues, the participant will be kindly requested to put your signature of the agreement. Your signature indicates that the participant is voluntary to participate in the study.

If you have any question or problems please contact the following address:

Principal Investigator: Mr. Hagos H/slassie (Bsc)

Mobile Phone: +251914404630

Email: hagoshaileslasie78@gmail.com

Advisors:

Dr. Aster Tsegaye (Msc, PhD)

Mobile Phone: +251911696085

Email: tsegayeaster@yahoo.com

Department of Medical Laboratory Science Research and Ethics Committee office

Telephone: +251 11275 5170 37

**Certificate of assent**

I am voluntary as I have communicated with my guardian/parent about the previous information, or as I have read it. I have had the opportunity to ask questions about it and any questions that I have asked have been answered to my satisfaction. My child is assented voluntarily to participate in your research.

Signature of Participant _______________

Date ___________________________Day/month/year

**Statement by the researcher**

I confirm that the participant was allowed to ask questions about the study, and all the questions asked by the participant have been answered correctly and to the best of my ability. I confirm that the parents/guardians haven’t been coerced into giving assent, and the assent has been given freely and voluntarily.

Name of guardian/parent taking the assent________________________

Signature of guardian /parent taking the assent____________________

Date ___________________________

Day/month/year

**Annex II: Questionnaire (English version)**

**Questionnaires to be filled by health professionals**

**Part I. General information**

Code Number__________________ Region _______________ Zone __________________

Sub city _____________________ Kebele _________________

**Part II. Personal information**

1. Age (in years) _____________________
2. Sex ________

|  | **History of common diseases** | |  |
| --- | --- | --- | --- |
| 3. | History of diabetes | | 1. Yes 2. No |
| 4. | History of Hypertension | | 1. Yes 2. No |
| 5. | History of Blood transfusion for the last 1 year | | 1. Yes 2. No |
| 6. | History of Hospital Admission for the last 1 year | | 1. Yes 2. No |
| 7. | History of Surgical procedure for the last three years? | | 1. Yes 2. No |
| 8. | History of chronic gastritis | | 1. Yes 2. No |
| 9. | History of Malaria for the last 6 months | | 1. Yes 2. No |
| 10. | History of TB for the last two years | | 1. Yes 2. No |
| 11. | History of Cancer | | 1. Yes 2. No |
| 12. | History of Cardiac illness | | 1. Yes 2. No |
| 13. | History of Bleeding disorders | | 1. Yes 2. No |
| 14. | History of allergy | | 1. Yes 2. No |
| 15. | History of Wheezing | | 1. Yes 2. No |
|  | **Part III. Anthropometric measurement** | | |
| 16. | Height (in cm) | ___________ | |
| 17. | Weight (in kg) | ___________ | |
| 18. | MUAC | ______________in cm ( will be interpreted later) | |
| 19. | Blood pressure (mm Hg) | ___________ | |

**NB**: If a participant answers **yes for one of the** questionnaire from No_-_ 3 to No_-_ 15 and the blood pressure is out of **90-120 systolic** and **60-90 diastolic** except with some preconditions they cannot include in the analysis.

- We thank you for your cooperation!

Interview Date:__________________

Interviewer’s Name_________________

Signature_________________

**Annex III: Questionnaire Amharic version (ቃለመጠይቅ** **12—17 ዓመት ለሆኑ ህፃናት መረጃ)**

**የፕሮጀክቱ ርዕስ፡**“እድሜአቸው ከ12-17 ዓመት ለሆኑ የመቀለ ነዋሪዎችየጤናማ ሰው ደም ውስጥ የሚገኙ ምርመራዎች መጠን ሪፈረንስ ኢንተርቫል እና በላቦራቶሪ ውስጥ የጥራት መመርመሪያ ንጥረ ነገር መስራት ነው፡፡

**የጥናቱ ተመራማሪ:** ሓጎስ ሃይለስላሰ ወልደሃወርያት

**መግቢያ:**

ጤና ይስጥልኝ! ስሜ ሓጎስሃይለስላሰእባላለሁ፡፡ የኣዲስ ኣበባ ዩኒቨሪስቲ የድህረ ምረቃ ተማሪ ሲሆን የመመረቂያ ፁሑፌን በሄማቶሎጂ ምርመራዎች መጠን ሪፈረንስ ኢንተርቫል እድሜአቸው ከ 12-17 ዓመት ለሆኑ የ መቀለ ነዋሪዎች እየሰራሁ ነው፡፡

**የምርምር ጥናቱ አላማ:**

የህክምና ላቦራቶሪ በጤናው አገልግሎት ውስጥ ከፍተኛ ሚና ይጫወታል፡፡ ምርመራን ለማረጋገጥ፣ ህሙማን ለመድሃኒቶች ምላሽ መስጠታቸውን ክትትል ለማድረግ፣ የበሽታዎችን ስርጭት ለማጥናት፣ በሽታ ለመከላክል እና ስለበሽታዎች ምንጭ ምርምር ለማድረግ አስተዋፅዎ ያደርጋል፡፡ በተለይም በአገራችን የጤናማ ሰው የላቦራቶሪ ውጤት ማመዳደሪያ ሪፈረንስ ኢንተርቫል እና በአገር ውስጥ የሚመረት የጥራት መመርመሪያ የለም፡፡ ስለሆነም የዚህ ጥናት ዓላማ በአገር ውስጥ በላቦራቶሪ ውስጥ የሚመረት የጥራት መመርመሪያ እናየጤናማ ሰው የሄማቶሎጂ ውጤት ማወዳደሪያ ሪፈረንስ ኢንተርቫል መሥራት ነው፡፡

አንተም/አንቺም በዚህ ጥናት እንድትሳተፍ/ፊ እየጋበዝን ወላጆችሽ/ወላጆችህ ፈቃዳቸውን ገልፀዋል፡፡፡ ስለዚህ በዚህ ጥናት በመሳተፍ በአገራችን በላቦራቶሪ ውስጥ የሚመረት የጥራት መመርመሪያ እናየጤናማ ሰው የክሊኒካል ላቦራቶሪ ውጤት ማመዳደሪያ ሪፈረንስ ኢንተርቫል ለመስራት አስተዋፅዎ እንድታደርግ/ጊ ተጋብዘሃል/ሻል፡፡ ሁለቱም ጥራት ያለው የላቦራቶሪ አገልግሎት ለመስጠት አስፈላጊ ናቸው፡፡

**የጥናቱ አካሄድ:**

በጥናቱ ለመሳተፍ ከተስማማህ/ሽ የጥናቱ አባል/አባላት 10 ደቂቃ የሚወስድ ጥያቄ ይጠይቁሃል/ሻል፡፡ ክብደት፣ ቁመት፣ የክንድ እና የደም ግፊት ልኬት ይወሰዳል፡፡ ሽንትና አይነምድር በምንሰጠው እቃ እንድትሰጠን/ጭን እንጠይቃለን፡፡፣ በተጨማሪም 4 ሚሊ ሊትር (ግማሽ የሾርባ ማንኪያ የሚሆን) በንፁህ ቫኩቴይነር ብልቃጥ እና መርፌ ፡፡ፓራሲቶሎጂ እና በሄማቶሎጂምርመራዎችን እናካሂዳለን፡፡

**ሚስጥር ስለመጠበቅ:**

በዚህ ጥናት የሚሰበሰብ መረጃ በሙሉ በሚስጥር ይጠበቃል፡፡ መረጃ በዚህ የስምምነት ቅፅ ከተፈቀደው ውጪ ለሶስተኛ ወገን ተላልፎ አይሰጥም፡፡ የዚህ ጥናት ውጤት ሊታተም ይችላል ነገር ግን የጥናቱ ተሳታፊዎች ስምና ማንኛውም መለያ አይገለፅም፡፡ ሚስጥራዊነቱን ለመጠበቅ የዚህ ጥናት አባላት መረጃዎችን በተቆለፈ ክፍል በተቆለፈ ካቢኔት ውስጥ ያስቀምጣሉ፣ የፈቃደኛ ተሳታፊዎችን ማንነትን ላለማሳወቅ ውጤቶችም በኮድ ይቀመጣሉ፡፡ በኮምፒዩተር ውስጥ ለተቀመጡ ፋይሎች ለጥናቱ ተመራማሪዎች ብቻ የሚፈቀዱና በሚስጥር ቁልፍ የሚጠበቁ ይሆናል፡፡ የተሳታፊ ውጤት ለህክምና ባለሞያ ሊተላለፍ የሚችለው በተሳታፊው ፈቃድ ብቻ ነው፡፡ የተሰበሰበው ሽንት፣ ዓይነምድርና ደም ለሌላ አገልግሎት አይውልም፡፡ በመጨረሻም ተሰርቶባቸው የተራረፉ የሚደፉ ናሙናዎች አካባቢን በማይበክል መልኩ በጥንቃቄ ይወገዳሉ፡፡

**ጥናቱ የሚያስከትላቸው የጤና ችግሮችና አለመመቸት:**

ሽንትና ዓይነምድር በመስጠት የሚደርስ መጠነኛ አለመመቸት ሊኖር ይችላል፡፡ ሆኖም ደም በሚቀዳበት ጊዜ መጠነኛ መጎዳትና የተወሰነ አለመመቸት ሊኖር ይችላል፡፡ ይሁን እንጂ በተቻለ መጠን ልምድ ያለው የላቦራቶሪ ባለሞያ በመጠቀም አለመመቸቱን ለመቀነስ እንሞክራለን፡፡

**ደህንነት:**

የደም ናሙና በሚወሰድበት ጊዜ በንፁህ የደም መቅጃ በመጠቀም የሚቀዳውን ቦታ በ70% አልኮል በማፅዳት ልምድ ባለው ባለሞያ ይከናወናል፡፡ በተጨማሪም ጥቅም ላይ ከዋሉ በኋላ ለማስቀመጥ የማይሆኑ የሚደፉ የዓይነምድር፣ ሽንት እና ደም ትራፊዎች የላቦራቶሪ ደህንነት መመሪያ በመከተል ይወገዳሉ፡፡

**ጥቅማ ጥቅሞች፡**

በዚህ ጥናት በመሳተፍ ለበሽታ አምጪ ተህዋስያን፣ ደምና ሽንት ምርመራ በማድረግ የጤንነት ሁኔታ ማወቅ ይቻላል፡፡ በአገር ውስጥ በላቦራቶሪ ውስጥ የሚመረት የጥራት መመርመሪያ እናየጤናማ ሰው የሄማቶሎጂ ውጤት ማመዳደሪያ ሪፈረንስ ኢንተርቫል ለማሻሻል ይረዳል፡፡

**በጥናቱ ለመሳተፍ ማትጊያ:**

ከዓይነምድር፣ ሽንት እና ደም ምርመራ ጤናማ ያልሆነ ውጤት ከተገኝ በአቅራቢው ወደ ሚገኝ ጤና ተቋም ትላካለህ/ትላኪያለሽ፣ የላቦራቶሪ ውጤቶቹን በነፃታገኛለህ/ታገኚያለሽ፡፡ ይሁን እንጂ በዚህ ጥናት ለመሳተፍም ሆነ ለመድሃኒት ክፍያ አይሰጥም፡፡ ስለተሳትፎህ/ህ ግን እናመሰግናለን፡፡

**ያለመሳተፍ መብት:**

በዚህ ጥናት ከተሳተፍክ/ሽ የቻልነውን ሁሉ እንክብካቤ እናደርጋለን፡፡ በማኛውም ሰዓት ከጥናቱ መውጣት እንደሚቻልና ይህም በምታገኘው/ኚው አገልግሎት ላይ (ለምሳሌ የጤና አገልግሎት) ምንም አይነት ልዩነት አይደረግም፡፡

**ጥያቄ ካለ ለማነጋገር:**

ምንም ዓይነት ጥያቄ ካለ የዓይነምድር፣ ሽንት እና የ ደም ናሙና የሰጠኸውን/የሰጠሽውን ሰው መጠየቅ ይቻላል፡፡

**በጤና ባለሞያዎች የሚሞላ ቃለ-መጠይቅ**

**መመሪያ**

በቅድሚያ ይህንን ቃለ-መጠይቅ ለመሙላት ለሰጡን ጊዜና ትብብር አድናቆቴን እገልፃለሁ፡፡ የዚህ ቃለ-መጠይቅ አላማ “በላቦራቶሪውስጥየጥራትመመርመሪያንጥረነገርእናየጤናማሰውደምውስጥየሚገኙየሄማቶሎጂምርመራዎችመጠንሪፈረንስኢንተርቫልእድሜአቸውከ 12-17ዓመት ለሀኑ የመቀለ ነዋሪዎችለመስራትመረጃለመሰብሰብነው፡፡የዚህጥናትሃሳቡንያመጡትየጥናቱ ተመራማሪ **ሓጎስ ሃይለስላሰ** በአዲስ አበባ ዩኒቨርስቲ የህክምና ላቦራቶሪ ትምህርት ክፍል የድህረ ምረቃ ተማሪ ሲሆኑ የመመረቂያ ፅሁፋቸው ሊሰሩበ ነው፡፡ስለሆነም የእርስዎ ቅን ትክክለኛ መልስ በሰዓቱ መስጠት የዚህን ጥናት ስኬት ይወስናል፡፡

አመሰግናለሁ!**!!**

**ክፍል 1.አጠቃላይመረጃ**

ኮድ__________________ ክልል _________________ ዞን__________________

ከተማ/ክፍለከተማ _____________________ቀበሌ _________________

**ክፍል 2.የግልመረጃ**

1. እድሜ _____________________
2. ጾታ________

|  | **የሚከተሉትየህመምዓይነቶችአሞዎትያውቃል?** |  |
| --- | --- | --- |
|  | የስኳርህመም? | 1. አዎን 2. የለም |
|  | የደምግፊትከፍማለት? | 1. አዎን 2. የለም |
|  | ባለፈው 1 ዓመትደምተሰጥቶዎያውቃል? | 1. አዎን 2. የለም |
|  | ባለፈው 1 ዓመትሆስፒታልተኝተውያውቃሉ? | 1. አዎን 2. የለም |
|  | ባለፉት 3 ዓመታትየቀዶህክምናተደርጎልዎያውቃል? | 1. አዎን 2. የለም |
|  | የቆየየጨጓራህመምአለብዎት? | 1. አዎን 2. የለም |
|  | ባፉት 6 ወራትየወባህመምአጋጥሞዎትያውቃል? | 1. አዎን 2. የለም |
|  | ባለፉት 2 ዓመታትየቲቢህመምኖሮዎትያውቃል? | 1. አዎን 2. የለም |
|  | ካንሰርህመም | 1. አዎን 2. የለም |
|  | የልብህመም | 1. አዎን 2. የለም |
|  | የመድማትችግር/ህመም | 1. አዎን 2. የለም |
|  | አለርጂ (የሰውነትመቆጣት) | 1. አዎን 2. የለም |
|  | የመተንፈስችግር (ሲተነፍሱሲርሲርየሚልድምፅ) | 1. አዎን 2. የለም |

|  | **ክፍል 4. ክብደት፣ቁመት፣የክንድና የደም ግፊት ልኬት** | |
| --- | --- | --- |
|  | ቁመት | ___________ሴንቲሜትር |
|  | ክብደት | ___________ኪሎግራም |
|  | የክንድመሃለኛውክፍልዙሪያው (MUAC) | ______________ሴንቲሜትር |
|  | የደምግፊት (በሚሊሜትርሜርኩሪ) | ___________(mm Hg) |

**ማሳሰብያ፡ ከተራ ቁጥር 3-15 መልስዎ አዎን ከሆነ ከጥናቱ ዉጪ ይሆናሉ**

- ስለትብብርዎእናመሰግናለን!

ቃለመጠይቅየተደረገበትቀን:__________________

ቃለመጠይቁንያካሄደውስም_________________ ፊርማ_________________
