# Supplementary material for: Community based hematological reference intervals among apparently healthy adolescents aged 12-17 years in Mekelle city, Tigrai, northern Ethiopia: A cross sectional study
Source: PLoS One. 2020 Sep 14;15(9):e0234106. doi: 10.1371/journal.pone.0234106 (PMC7489559; doi:10.1371/journal.pone.0234106)
Supplement: Attachment — Submitted filename: Response to Reviewers.docx [file pone.0234106.s003.docx]

Mr. Hagos Haileslasie Weldehaweriat

Hematology and Immunohematology Unit
Department of Medical Laboratory Science

College of Medicine and Health Sciences
Adigrat University
P.O. Box 50
Ethiopia

Telephone +251-914404630
[hagoshaileslasie78@gmail.com](mailto:hagoshaileslasie78@gmail.com)

Dear Editors

I am very happy to resubmit again for publication the revised manuscript entitled “Establishment of community based hematological reference intervals among apparently healthy adolescents aged 12-17 years in Mekelle city, Tigrai, northern Ethiopia: a cross sectional study 2019**’’.**

Thank you for such a wonderful contribution for editing and review the manuscript. I value the insights and guidance you provide for the manuscript. I believe that all of your advice and comments are very important for scientific quality of the manuscript. I attempted to understand your requested comment and general instructions for the manuscript. Mainly we have tried to touch the main comment raised by the editors and reviewers. Therefore, the manuscript was revised again based on editors and reviewers comments and advice.

We believe our finding will helps readers of PLOS ONE JOURNAL mainly on hematological reference intervals. We need to confirm to you this manuscript has not been previously published. In addition all of the authors have approved the contents of this revised manuscript and have agreed for resubmission to PLOS ONE journal.

Sincerely,

Hagos Haileslasie Weldehaweriat (MSc)

**Point by point response for reviewers**

**Response for reviewer 1**

**Comment 1**: Please review the manuscript throughout with a fluent English speaker.

**Response:** The grammatical and writing errors are corrected. The manuscript was drafted and rewrites again.

**Comment 2:**  Median age (abstract): report as XX.X, the extra level of precision (15.31) is unnecessary

**Response:** The decimal number are corrected in the manuscript and rewritten again
**Comment 3:** How many outliers did you exclude? Should you be doing this?

Response: Yes, 20(5.8%) of the participants were excluded due to its outlierness. Please, see on page 9 on the **result** section.

**Comment 4:** Please clarify where and why you combined values. I presume this was because they were not

Significantly different? Is this mentioned in the methods?

**Response:** No, We included in the new revised manuscript in the result part **page 10**.

**Comment 5:** what are “the company’s reference intervals (Table 3)”?

**Response:** The value which is inserted on the machine using the values of the test leaflet of the specific machine. When a new machine come to install in a Laboratory the machine comes with its own specific leaflets. Inserted the values to the machine to use as a reference

**Comment 6:** I recommend ending your introduction with a quick statement about what you propose to present in this paper

**Response:** We accept your comment and the manuscript is modified. You can see at the last of the introduction part.

**Response for reviewer 2**

**Comment 1**: I assume that presently RIs from the manufacturer is used. Company values are stated in several tables. Where these figures for adults, or for adolescents, or both? This is needed to evaluate how these new RIs compare to the presently RIs used.

**Response:** The inserted values on the CBC machine were for both adolescents and adults. There was no separated value on the machine but I compare the result with the leaflet which I have got separate values for adolescents and adults in the manual CBC machine**.**

**Comment 2:** Abstract: could be shortened, omit spelling out EDTAs chemical name, what statistical software was used. Also, numbers for RIs could be omitted, but hematology parameters measured should be included, along with a brief description of gender differences.

**Response:** We abbreviate the long terms of EDTA and numbers out of the hematological parameters. The data were entered and analyzed by SPSS
version 23 statistical software.

**Comment 3:** Background, page 3, last paragraph. I assume studies on pharmaceuticals are meant when stating “toxicity grading scales”, but these sentences are unclear.

**Response:** During the treatment of hematological cases for the patients it is based on the WHO guideline which prepared based on the developed countries. So, it is better to follow clinical cases of the patients in relation to the local population.

**Comment 4:** Methods: 3.1 could be shortened, e.g. “Mekelle was formerly the capital”, “Mekelle is considered a special zone”. Weyna-Dega climate and altitude is relevant, but preferably state something about this climate instead of using a local term

**Response:** We tried to shortened and local terms are avoided

**Comment 5:** CLSI guidelines should be accompanied by a reference.

3.3 including table could be shortened.

**Response:** Tables are slightly modified and CLSI reference is putting on the fifth line of the 3.2 sample size determination part [2, 4].

**Comment 6:** 3.4 How many adolescents did not give assent to participate? Such figures are needed to know how representative this population was when using this random enrollment design

**Response:** All of the participants who were present at study area during data collection time and who fulfilled the criteria were voluntarily participated after oriented the aim of the research.

**Comment 7:** 3.6 Samples were transported ice-cooled within “allowable time”? What was the maximal time allowed until analysis?

**Response:** The maximum allowable time was 5 hours. I have corrected on the manuscript part

**Comment 8:** During how long period was the collection of samples performed? Hematology systems sometimes drift over time.

**Response:** The data collection was performed from the middle of December to the last of April within 4 months.

**Comment 9**: Where there any internal or external controls used to assure stability over time for the measurements, and comparability to other labs?

**Response:** Yes**,** We have used both internal and external controls. Comparability was done between Ayder referral Hospital hematology CBC value and Wukro General Hospital. The result was similar related to each other by transporting the sample within 5 hours like that of participant’s sample.

**Comment 10:** 3.8 CLSI suggests Harris Boyd (or Lahti) statistics for partitioning. In the study Mann Whitney U-test was employed. What considerations were behind that decision?

**Response:** Harris Boyd (or Lahti) is important for partitioning of the study participants but Mann Whitney U-test is used for analysis of the data to compare the gender difference between males and females based on their median values.

**Comment 11:** Ethical considerations. Some countries do not allow recording of religious belief in scientific studies, as could be seen in Table 2. Was religion covered by the ethical clearance?
**Response:** No, because in Ethiopia many research are done without religion ethical clearance. There is no problem participating a religion belief in scientific studies.

**Comment 12:** Operational definitions could be omitted, and the information in that section integrated into the Methods section.

**Response:** we have omitted and merge to the methodology parts

**Comment 13:** Results: Figure 1 shows age, however I cannot find any fig 1.The 1st paragraph relating figures for numerous RIs could be shortened; figures could be omitted or substantially shortened since they also appear in Table 3. Now table 3 shows combined only for those three parameters where the Mann Whitney U-test turned out with insignificant differences between genders (MCH, Neutrophils, lymphocytes in %). However, even though lymphocyte% is insignificant (0.48), lymphocyte in absolute numbers is highly significant (0.004). Thus, I suggest the authors show all data for males and females as well as combined.
Page 12 starts with “Comparing the upper and lower limits….” I assume the authors refer to
Table 4 not to Table3

**Response:** Figure and Tables rearranged and corrected the problem was due to typical errors.

**Comment 14:** Table 5 is a comparison of RIs from different sources. The United States/Europe lack reference/s Also, information about ages is relevant for these studies, e.g. in the Ethiopian children from the age of 5 years are compared to the present study. This is a major limitation in the presentation; comparisons to gender and age in other studies, and to RIs presently used are difficult to follow. Thus, the practical use even in the area where the study was conducted is unclear.

**Response:** We put the reference for United States/Europe on the appropriate space on **Table5**.
