# Supplementary material for: Community based hematological reference intervals among apparently healthy adolescents aged 12-17 years in Mekelle city, Tigrai, northern Ethiopia: A cross sectional study
Source: PLoS One. 2020 Sep 14;15(9):e0234106. doi: 10.1371/journal.pone.0234106 (PMC7489559; doi:10.1371/journal.pone.0234106)
Supplement: Attachment — Submitted filename: Response to Reviewers 3.docx [file pone.0234106.s004.docx]

Mr. Hagos Haileslasie Weldehaweriat

Hematology and Immunohematology Unit
Department of Medical Laboratory Science

College of Medicine and Health Sciences
Adigrat University
P.O. Box 50
Ethiopia

Telephone +251-914404630
[hagoshaileslasie78@gmail.com](mailto:hagoshaileslasie78@gmail.com)

Dear Editors

I am very happy to resubmit again for publication the revised manuscript entitled “Community based hematological reference intervals among apparently healthy adolescents aged 12-17 years in Mekelle city, Tigrai, northern Ethiopia: a cross sectional study 2019**’’.**

Thank you for such a wonderful contribution for editing and review the manuscript. I value the insights and guidance you provide for the manuscript. I believe that all of your advice and comments are very important for scientific quality of the manuscript. I attempted to understand your requested comment and general instructions for the manuscript. Mainly we have tried to touch the main comment raised by the editors and reviewers. Therefore, the manuscript was revised again based on editors and reviewers comments and advice.

We believe our finding will helps readers of PLOS ONE JOURNAL mainly on hematological reference intervals. We need to confirm to you this manuscript has not been previously published. In addition all of the authors have approved the contents of this revised manuscript and have agreed for resubmission to PLOS ONE journal.

Sincerely,

Hagos Haileslasie Weldehaweriat (MSc)

**Point by point response for reviewers**

**Response for reviewer 3**

**Comment 1:** I suggest the title to be adjusted as “Community based hematological reference intervals among apparently healthy adolescents aged 12-17 years in Mekelle city, Tigrai, Northern Ethiopia: a cross-sectional study”

**Response 1:** We have changed the Title to “Community based hematological reference intervals among apparently healthy adolescents aged 12-17 years in Mekelle city, Tigrai, Northern Ethiopia: a cross-sectional study”

**Comment 2:** Topographical errors throughout the manuscript: spelling, capitalization of proper nouns including author names, sentence form (present Vs past) and grammatical problems

**Response 2:** We have corrected the topographical errors

**Comment 3:** There are sentences that start with numbers here and there. It is not recommended to start a sentence with numbers please correct.

**Response 3:** We have corrected it (Example: Summary Result part)

**Comment 4:** Information which is not related to the manuscript should be removed e.g. in the background section paragraph 2 last 3 sentences have low value to the manuscript.

“Growth is an extremely complex and non-linear biological process, driven by hormonal mechanisms, characterized by an intrinsic variability reflecting environmental, genetic influences and individual adaptive responses. Growth charts are very helpful as reference tools for pediatricians, in order to monitor individual growth and provide therapeutic interventions. Most female’s puberty starts around 12 years but males from 14 years [6].”

**Response 4:** We have tried to remove the unwanted part and tried to rearrange it.

**Comment 5:** Repetitions: e.g. last two sentences of Paragraph 4 of Background: please merge them. Adult Africans have been shown to have lower levels of hemoglobin, red blood cells, platelets, and neutrophil counts compared to adults in the developed countries [12-15]. Further, most blood specimens of Africans have been found to have lower Hemoglobin (Hgb), Hematocrit (Hct), Red blood cell count (RBC), mean corpuscular volume (MCV), neutrophil and platelet counts [16].

**Response 5:** The unwanted repetition tried to merged and rearranged on **Paragraph 4** the Background part

**Comment 6:**	“Sex” should be replaced by “Gender”

**Response 6:** We tried to replace Sex by Gender (Summary background part, background section paragraph 2, discussion parts and also in other parts)

**Comment 7:** “Sampling technique” should be “Reference population selection technique”

**Response 7:** We have tried to replace “Sampling technique” by “Reference population selection technique”

**Comment 8:** “K” should be specified: what is the value of “K” it should be mentioned

**Response 8:** “K” is the interval of data taking households from the first household to the next (second) household. K^th^=total number of households in the 3 sub cities divided by total number of participants. K^th^=199 the participants data were collected every 199^th^ household.

**Comment 9:** Words such as “doesn’t” should be written in full term as “did not”

**Response 9:** We tried to corrected such like terms (discussion Paragraph 3)

**Comment 10:** Table 1 is not cited

**Response 10:** The data was obtained from the municipality of Mekelle city (unpublished data).

**Comment 11:** Were there significant differences in RIs of the three kebeles? And also, please clarify kebele? What do you mean by Kebele?

**Response 11:** There was no significance difference in RIs among the three kebeles. Kebele is part of a sub city which is a small administrating part of the government.

**Comment 12:** Blood sample for hemoparasites, Stool specimens for intestinal parasites examination and urine samples for urinalysis were collected but there is no lab analysis section. There must be laboratory analysis section for hemoparasite examination, Stool examination and urinalysis. A more complete description of the laboratory methods, especially metrological traceability, is required.

**Response 12:** We have tried to incorporate the Lab analysis section

**Comment 13:** How do you exclude the influence of infectious diseases such as HIV, Hepatitis on RIS?

**Response 13:** The health extension professionals have a family folder which follows the health status continuously. So, by following stringent criteria for exclusion before sample collection and those apparently healthy individuals sample was screened in the Tigrai blood bank using an ELISA technique.

**Comment 14:**	How representative is the population for their values to be used for reference intervals?

**Response 14:** The participants were 100% representative for the city because of their systematically selection methods. The three sub cities were selected randomly from the total of seven sub cities. According to CLSI guideline in order to say representative it is better to cover more than 30% of the total subjects.

**Comment 15:** Quality control: What was the validation status of the equipment and what QA protocols (maintenance, reproducibility, accuracy, between run, within run, etc) used to validate the analyzer used? These establish how accurate the analyzers’ used produce results. How long did samples stay before analysis and how were they kept in case samples were not analyzed immediately?

**Response 15:** The Hospital was on the process of accreditation. Its maintenances protocol was controlled according to its standard procedures continuously. Accuracy was done by using the daily QC tests (Low, Normal and High values) and also reproducibility was checked. Between run and within run also checks to avoid carry over between former and later samples. The sample was preserved within 2-8^o^c cold chain box until analysis. The CBC sample was done with in five hours of collection. The 2-8^o^c preserved sample was put in the room temperature for 30 minutes before analysis.

**Comment 16:** A comment should be made on the pre-analytical factors. Especially given the dependence of cell counts on haemoconcentration, it is necessary to indicate whether subjects are seated or lying, how long a tourniquet was applied for and also how long between collection and analysis.

**Response 16:** During the blood collection time participants was seated on a comfortably chair before collection and tourniquet was applied for less than one minute until the vein is visible and anchored with the syringe. One’s the vein is visible and anchored with the syringe the tourniquet was removed. The collected sample was analyzed with in five hours.

**Comment 17:** Data analysis: The statement “Data lower than first quartile (Q1-1.5 × IQR), or higher than third quartile (Q3+1.5 × IQR) (Whisker and blot method) were considered as outliers and the outlier was excluded.” Needs reference

**Response 17:** We have putting the reference for the outliers on the data analysis part

**Comment 18:** Data analysis: Do you check normality of data? Was the data normally distributed?

**Response 18:** Yes, We have checked the normality before doing the analysis. But, the data was not normally distributed.

**Comment 19:**	Results: Socio demographic characteristics: The use of mean and SD to describe the age distribution is inappropriate as the population age is clearly not Gaussian. I would recommend median, range and interquartile ranges or similar.

**Response 19:** We take the comments and corrected it.

**Comment 20:**	Result: The numbers in the statement “(15) were outliers (20) and presences of hemolysis (10”) are not clear

**Response 20:** We have tried to make clear and easily understandable

**Comment 21:**	Table 2 should be deleted. It has no value.

**Response 21:** Table 2 is removed completely

**Comment 22:**	Mean and 95% CI should be deleted from table 3 because it is inappropriate as the parameters value are clearly not Gaussian. I recommend the use of Median and Interquartile range.

**Response 22:** Ok, Mean is removed from the Table but 95% CI is important for RIs than the Inter Quartile range

**Comment 23:**	Table 5: should also include other studies, and also check reference 22, is it for US/Europe or Tanzanian?

**Response 23:** It is for Tanzanian it is a typical error we have corrected it

**Comment 24:**	Result and Discussion: Please remove comparisons that made with adult population. Some studies used for comparison are adult population RIs.

**Response 24:** We have tried to reject the comparison with adults

**Comment 25:** Discussion: Reference 22 and 23 are about RIs but how they could be a reference for the last statements in the first paragraph of the background?

**Response 25:** We have replaced and rearranged the unnecessary references

**Comment 26:**	Discussion: paragraph 4 and 5: The references should be revised…..

**Response 26:** Discussion: paragraph 4 is tried to rearranged with other references
