# Supplementary material for: Community based hematological reference intervals among apparently healthy adolescents aged 12-17 years in Mekelle city, Tigrai, northern Ethiopia: A cross sectional study
Source: PLoS One. 2020 Sep 14;15(9):e0234106. doi: 10.1371/journal.pone.0234106 (PMC7489559; doi:10.1371/journal.pone.0234106)
Supplement: Attachment — Submitted filename: Response to Reviewers.docx [file pone.0234106.s005.docx]

**Point by point response for reviewers**

**Response for reviewer 3**

**Comment 1:** Table 1 is not cited in the text

**Response:** We have tried to put the citation under the Table 1 but the number of participants from each Sub city and kebelles was obtained from the Mekelle municipality [unpublished data]

**Comment 2:** The comments not incorporated in the method section

**Response:** Comment 11 is included in the middle and last part of the reference population selection technique and data analysis part.

Comment 13 is included in middle part of the sample collection and Laboratory analysis section

Comment 15 is included in the data quality control section

Comment 16 is included in the sample collection and laboratory analysis section

Comment 18 is included in the data analysis section line 2

**Comment 3:** Table 3 needs modification

**Response 3:** Table 3 is modified and rearranged according to the given orientation

**Comment 4:** Table 2: partition in gender is not needed for parameters which have no significance difference b/n male and female.

**Response 4:** We have tried to avoid partitions for the parameters which have no significance difference b/n male and female in Table 2 and other Tables.

**Comment 5:** Table 2 Mixed value needs definition

**Response 5**: Since the machine was displayed the values of Eosinophiles, Basophile and Monocytes as mixed value due to its 3 differential nature of the machine. Definition putted under **Table 2**

**Comment 6:** Discussion: As indicated in the result section, most of the parameters show a statistically significant difference b/n Males and Females. There should be a comment on statistically Vs clinically significant difference

**Response 6:** If the p value is <0.05 there is significance difference b/n Males and Females means that there is clinically difference among the gender. It needs separate reference interval for the gender since they are clinically different.

**Comment 7:** Mean should be removed

**Response 7:** Mean is removed from the Tables

**Comment 8:** Sex should be replaced by Gender

**Response 8**: In whole part of the manuscript sex is replaced by Gender
